# Mapping of citrullinated fibrinogen B-cell epitopes in rheumatoid arthritis by imaging surface plasmon resonance

**DOI:** 10.1186/ar3205

**Published:** 2010-12-23

**Authors:** Joyce JBC van Beers, Reinout Raijmakers, Lou-Ella Alexander, Judith Stammen-Vogelzangs, Angelique MC Lokate, Albert JR Heck, Richard BM Schasfoort, Ger JM Pruijn

**Affiliations:** 1 Department of Biomolecular Chemistry, Nijmegen Center for Molecular Life Sciences, Institute for Molecules and Materials, Radboud University, PO Box 9101, NL-6500 HB Nijmegen, The Netherlands; 2 Biomolecular Mass Spectrometry and Proteomics Group, Bijvoet Center for Biomolecular Research and Utrecht Institute for Pharmaceutical Sciences, Utrecht University and Netherlands Proteomics Centre, Padualaan 8, NL-3584 CH Utrecht, The Netherlands; 3 Medical Cell Biophysics Group, MIRA institute, University of Twente, PO Box 217, NL-7500 AE Enschede, The Netherlands; 4 IBIS Technologies B.V., Pantheon 5, NL-7521 PR Enschede, The Netherlands

## Abstract

**Introduction:**

Rheumatoid arthritis (RA) frequently involves the loss of tolerance to citrullinated antigens, which may play a role in pathogenicity. Citrullinated fibrinogen is commonly found in inflamed synovial tissue and is a frequent target of autoantibodies in RA patients. To obtain insight into the B-cell response to citrullinated fibrinogen in RA, its autoepitopes were systematically mapped using a new methodology.

**Methods:**

Human fibrinogen was citrullinated *in vitro *by peptidylarginine deiminases (PAD), subjected to proteolysis and the resulting peptides were fractionated by ion exchange chromatography. The peptide composition of the citrullinated peptide-containing fractions was determined by high resolution tandem mass spectrometry. The recognition of these fractions by patient sera was subsequently analyzed by imaging surface plasmon resonance on microarrays.

**Results:**

In total about two-thirds of the 81 arginines of human fibrinogen were found to be susceptible to citrullination by the human PAD2, the human PAD4 or the rabbit PAD2 enzymes. Citrullination sites were found in all three polypeptide chains of fibrinogen, although the α-chain appeared to contain most of them. The analysis of 98 anti-citrullinated protein antibody-positive RA sera using the new methodology allowed the identification of three major citrullinated epitope regions in human fibrinogen, two in the α- and one in the β-chain.

**Conclusions:**

A comprehensive overview of citrullination sites in human fibrinogen was generated. The multiplex analysis of peptide fractions derived from a post-translationally modified protein, characterized by mass spectrometry, with patient sera provides a versatile system for mapping modified amino acid-containing epitopes. The citrullinated epitopes of human fibrinogen most efficiently recognized by RA autoantibodies are confined to three regions of its polypeptides.

## Introduction

Rheumatoid arthritis (RA) is a common autoimmune disease, in which several autoantigens have been identified, including fibrinogen [1-3]. Fibrinogen consists of two copies of each of its three polypeptide chains α, β and γ [4]. Fibrinogen is involved in the clotting cascade, in which it is converted into fibrin, a process mediated by thrombin [5]. Autoantibodies against citrullinated proteins (ACPA) have been shown to be specifically associated with RA and are already present prior to disease onset [6]. Citrullination, the conversion of peptidylarginine into peptidylcitrulline, of the fibrinogen α and β chains generates antigenic targets for autoantibodies present in the serum and synovial fluid of RA patients [1,7].

**Figure 1:**
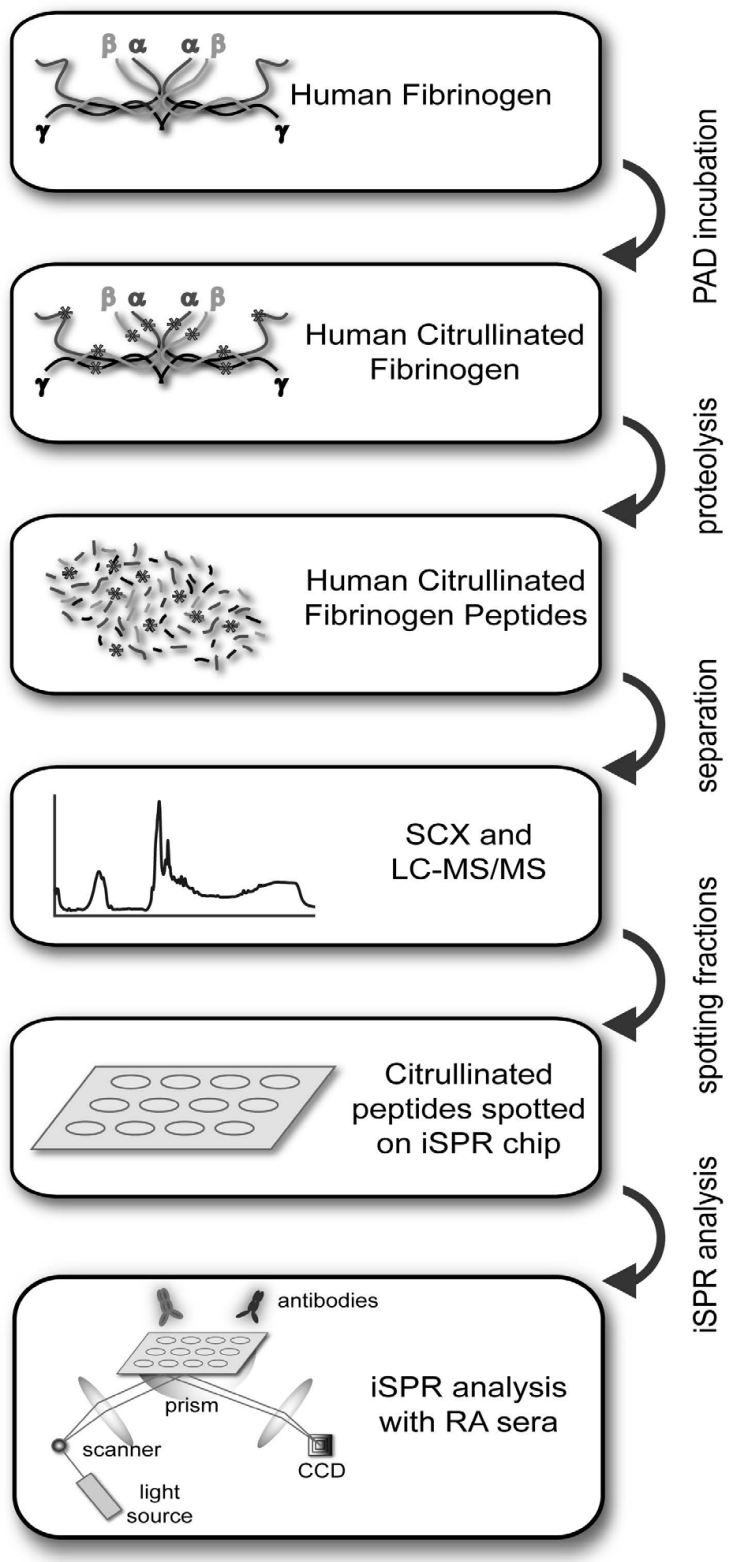
**Autoepitope mapping of citrullinated fibrinogen**. Schematic overview of the novel method used to characterize the citrullinated epitopes on human fibrinogen recognized by RA patient autoantibodies.

For the diagnosis of RA, besides the clinical symptoms, tests for detecting autoantibodies, such as rheumatoid factor (RF test) or ACPA (which are generally detected with the so-called cyclic citrullinated peptide, CCP, test) can be useful [8]. Autoantibodies to citrullinated human fibrinogen may have great value for the diagnosis of RA [9]. Vander Cruyssen and colleagues compared an anti-citrullinated fibrinogen ELISA with the anti-CCP test and detected similar diagnostic performance [10]. The role of citrullinated proteins and ACPA in the pathophysiology of RA is not fully understood, but it has been shown that citrullinated fibrinogen can induce arthritis in genetically susceptible (DR4-IE transgenic) mice [7]. Recently, Ho and others found that mice that were immunized with citrullinated fibrinogen developed arthritis and fibrinogen-reactive T cells which produce the proinflammatory cytokines IL-6, IL-17, TNF-α, and IFN-γ and that these mice possess rheumatoid factor, circulating immune complexes and anti-CCP, all of which are characteristics of human RA [11]. *In vitro *studies by Clavel and co-workers showed that immune-complexes consisting of ACPA and citrullinated fibrinogen can induce macrophage secretion of TNF-α, which is an important mediator of inflammation [12]. In humans, an association was detected between the occurrence of the RA susceptible HLA-DRB1 allele and the presence of anti-citrullinated fibrinogen antibodies [13]. Finally, circulating immune complexes containing citrullinated fibrinogen were found in a large subset of ACPA-positive RA patients [14]. These findings suggest a crucial role for fibrinogen in RA pathogenesis. Several studies have addressed the position of citrullinated autoepitopes in human fibrinogen [4,7,9,15,16]. Most of these studies were performed with synthetic citrullinated fibrinogen peptides in combination with ELISA detection. Here, we describe a novel method to map the epitopes of post-translationally modified proteins and apply this method, which is schematically illustrated in Figure 1, to map the autoepitopes of citrullinated fibrinogen recognized by RA sera.

## Materials and methods

### In vitro citrullination of fibrinogen

To generate deiminated fibrinogen, 1 mg of immunoglobulin-depleted human fibrinogen (Sigma-Aldrich, St. Louis, Missouri, USA) was *in vitro *citrullinated by either human PAD2 (hPAD2; 1 U), human PAD4 (hPAD4; 1 U) or rabbit muscle PAD (rmPAD2; 1 U; Sigma-Aldrich) in deimination buffer (40 mM Tris-HCl, pH 7.5, 5 mM CaCl_2 _and 10 mM DTT) and incubated at 37°C for three hours. After incubation, the reaction mixtures were dialyzed against phosphate-buffered saline overnight.

The recombinant hPAD2 and hPAD4 were expressed and purified as described previously [17]. The activity of both recombinant proteins was determined with a colorimetric assay [18,19].

### Western blot analysis

Human fibrinogen was separated by SDS-PAGE and transferred to nitrocellulose membranes. For the detection of citrullinated proteins, blots were chemically modified overnight as described previously [20]. After blocking the blots in blocking buffer (5% non-fat dried milk and 0.1% NP40 in PBS) for one hour at room temperature, they were incubated with the anti-modified citrulline antibody (Upstate, Billerica, Massachusetts, USA) in blocking buffer for three hours at room temperature. Finally, the blots were washed with blocking buffer and bound antibodies were visualized with an IRDye 800-labeled secondary antibody (Molecular Probes, Rockland Immunochemicals, Gilbertsville, Pennsylvania, USA) and monitored by infrared (LI-COR Odyssey, Lincoln, Nebraska, USA) imaging.

### Enzymatic digestion of citrullinated fibrinogen

Different proteases were used for the generation of citrullinated fibrinogen peptides to achieve optimal sequence coverage in mass spectrometry analyses and to obtain citrullinated epitopes in different sequence contexts for surface plasmon resonance imaging analyses.

Citrullinated fibrinogen was reduced using 50 mM DTT, alkylated in 100 mM iodoacetamide and digested with trypsin (all PADs), chymotrypsin (all PADs), Lys-N (rmPAD2 only), or GluC (hPAD2 and hPAD4), in a 1:100 ratio by overnight incubation at 37°C.

### Separation of fibrinogen peptide fragments

Peptides obtained by digestion of fibrinogen citrullinated by rmPAD2 were separated using strong cation exchange (SCX) chromatography, which was performed using an Agilent 1100 HPLC system (Agilent Technologies, Santa Clara, California, USA) with two C18 Opti-Lynx (Optimized Technologies, Oregon City, Oregon, USA) guard columns and a polysulfoethyl A SCX column (PolyLC, Columbia, MD, USA; 200 mm × 2.1 mm inner diameter, 5 μm, 200Å), essentially as described previously [21]. Ten percent of each fraction was analyzed by LC-MS/MS. Fractions containing a large overlap in citrullinated peptides were pooled prior to SPR analysis. This led to a total of 29 fractions, derived from either the chymotrypsin (C), Lys-N (L) or trypsin (T) digestion. Each selected fraction was desalted and dried prior to immobilization on the sensor disc surface.

### LC-MS/MS

Digested citrullinated fibrinogen samples were analyzed by nano-LC-MS/MS on an LTQ-Orbitrap-XL using electron transfer induced dissociation (ETD) (hPAD2 and hPAD4) and/or an LTQ-FTICR (all PADs) mass spectrometer using collision induced dissociation (CID) (Thermo, San Jose, CA, USA) connected to an Agilent 1200 series nano-LC system. Peptides were separated on C18 with a multi-step gradient of 0.6% acetic acid (buffer A) and 0.6% acetic acid/80% acetonitrile (buffer B). Raw MS data were converted to peak lists using Bioworks Browser software, version 3.1.1 (Thermon Finnigan, San Jose, California, USA) and searched with Mascot against the Swiss-Prot database (Swiss Institute of Bioinformatics, Lausanne, Switzerland), using the appropriate enzyme and allowing citrullination of arginine, deamidation of asparagine and oxidation of methionine as variable modifications. The spectra from the hPAD2 and hPAD4 fibrinogen incubations were searched with a precursor mass tolerance of 20 ppm and a product mass tolerance of 0.9 Da, allowing two miscleavages, with peptide identifications accepted with a Mascot score greater than 25. For the SCX separated rmPAD2 modified peptides, precursor tolerance was 5 ppm, product mass tolerance 0.9 Da, allowing one miscleavage and peptide identifications were accepted with a slightly lower Mascot score of at least 20 to be able to better identify overlap between adjacent fractions.

### Preparation of the microarrays and iSPR analyses

To prepare the fibrinogen peptides for immoblization on the surface plasmon resonance (SPR) sensor discs, it was chosen not to use the GluC protease, which is known for its incomplete and heterogeneous cleavage, making it not very suitable for this application. Instead, Lys-N was used, which cleaves at the N-terminal side of lysine residues, thereby generating peptides that would always be immobilized in the same orientation, because both primary amines available for crosslinking are located at the N-terminus of the peptide. In addition to LysN, trypsin and chymotrypsin were used to digest human fibrinogen citrullinated *in vitro *with rmPAD2. The latter PAD was used for these experiments, because its specific activity is higher than that of the recombinant human PADs, implying that lower quantities could be used, which reduces the presence of PAD-derived peptides in the citrullinated fibrinogen fractions.

Fibrinogen peptide fractions were immobilized on an *i*SPR chip containing a dextran hydrogel linked to a gold layer on a round glass surface (HC200, Xantec Bioanalytics GmbH, Dusseldorf, Germany). In short, immobilization of the fibrinogen peptide fractions was initiated by activation of the dextran hydrogel, which is achieved by creating a reactive N-hydroxysuccinimide ester with the carboxyl groups of the dextran matrix via the reaction which occurs when 0.8 M N-hydroxysuccinimide (NHS) and 0.2 M N-ethyl-N'-(dimethylaminopropyl) carbodiimide hydrochloride (EDC) are mixed and added to the sensor disc surface. After incubation for 20 minutes the sensor disc was washed with 0.25% acetic acid, pH 4.5 and dried under nitrogen for 30 minutes. The protease-digested, citrullinated fibrinogen SCX fractions, human IgG (to visualize the array) and control synthetic peptides (citrullinated peptides and their arginine-containing counterparts) were spotted on the surface of the sensor discs using a noncontact spotter (1 nl droplets with spotting concentration of 1 ng/nl in 50 mM MES, pH 5.4) and placed in a humidity chamber at room temperature for 1 h. Finally, the unreacted groups were blocked by incubation with 1 M ethanolamine, pH 8.0 for 10 minutes. The sensor discs were rinsed with PBS and kept wet until use.

The incubation with patient sera, and washing and regeneration of the coated sensor discs were performed as described previously [22,23]. In contrast to previous studies, a new valveless sample injection method in combination with high mass transport of analytes was used.

### Multiplex iSPR analyses of fibrinogen peptide recognition by patient sera

Sera from rheumatoid arthritis patients were obtained from the Department of Rheumatology, University Medical Center St. Radboud, Nijmegen, The Netherlands. The use of autoimmune patient sera for autoantibody studies has institutional review board approval and patients provided informed consent. Prior to use, sera were 50-fold diluted in PBS-0.03% Tween-20. Using the IBIS-iSPR instrument a diluted serum sample plug of 80 μl was injected and 20 μl was guided backward and forward over the microarray of fibrinogen peptide fractions in a flow cell with a speed of 30 μl/s to allow autoantibody binding to the immobilized peptides.

To obtain quantitative results for antibody binding to the peptide fractions, the data were analyzed by the SPRint software (IBIS Technologies BV., Enschede, The Netherlands). Spots were classified as being strongly or moderately recognized by sera when at least 25% of the sera were reactive with these fractions with angle shifts higher than the mean plus eight, respectively two, standard deviations of the angle shift observed for a number of anti-CCP-negative RA sera.

## Results

### Identification of citrullination sites in human fibrinogen

**Figure 2:**
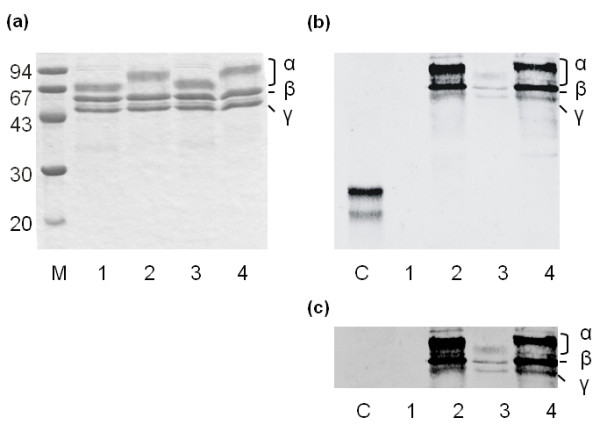
***In vitro *citrullination of human fibrinogen**. **(a) **Human fibrinogen (lane 1) was citrullinated *in vitro *with the recombinant hPAD2 (lane 2), hPAD4 (lane 3) and rmPAD2 (lane 4). The reaction products were analysed by SDS-PAGE and Coomassie Brilliant Blue staining (a) or by western blotting followed by detection of citrullinated proteins by anti-modified citrulline antibodies after modification of proteins on the blot **(b, c)**. (c) Represents a longer exposure of the upper region of the blot shown in (b). The positions of the α-, β- and γ-chains of fibrinogen are indicated. Lane M contains a molecular weight marker and the molecular weights of the marker proteins are indicated on the left. In (b) lane C contains deiminated soybean trypsin inhibitor as a positive control for the detection of citrullination.

To generate a map of the citrullinated autoantigenic epitopes on human fibrinogen, the protein was first citrullinated *in vitro *using recombinant hPAD2 and hPAD4, as well as rabbit muscle PAD (rmPAD2). To check the resulting citrullination of the protein, the reaction products were analyzed by SDS-PAGE and Coomassie brilliant blue staining. Bands corresponding to the α-, β- and γ-chains of fibrinogen were detected and upon incubation with PAD the migration of the α-chain and, although to a much lesser extent, that of the β-chain were changed (Figure 2a). To confirm that this migration shift was due to citrullination, blots containing the same samples were analyzed using the anti-modified citrulline antibody procedure (Figure 2b). The results showed that all fibrinogen chains were citrullinated by all PADs, although citrullination by hPAD4 appeared to be less efficient than that of the other PADs. In agreement with its migration shift, the α-chain displayed the highest levels of citrullination upon incubation with hPAD2 and rmPAD2. To exclude that the lower levels of fibrinogen citrullination by hPAD4 was due to differences in the specific activity of the PAD enzymes, the same amount of fibrinogen was incubated with increasing amounts of hPAD4 (up to 20 U/mg substrate). However, this did not lead to increased levels of citrullination (data not shown), suggesting that hPAD4 is more restrictive in citrullination of fibrinogen than hPAD2 and rmPAD2.

**Figure 3:**
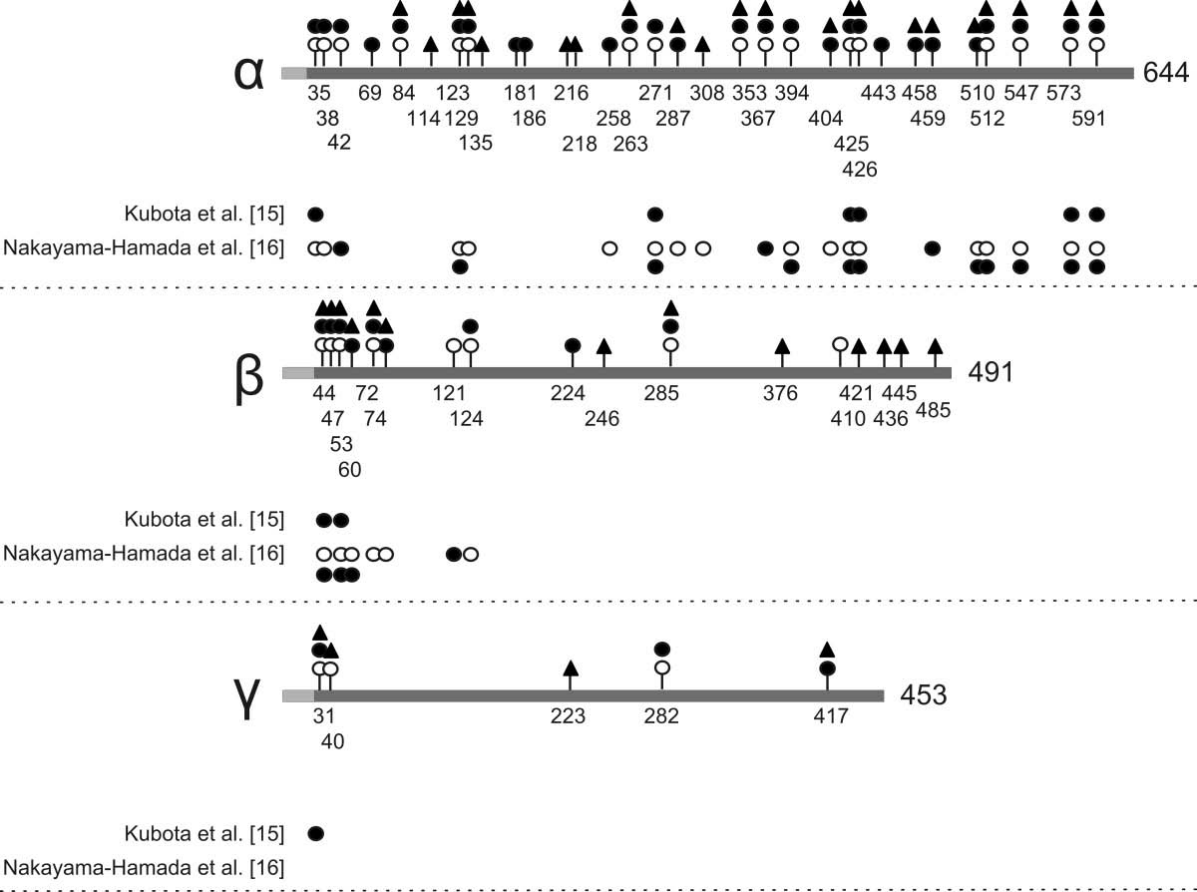
**Citrullination sites in human fibrinogen**. Human fibrinogen was citrullinated *in vitro *by hPAD2, hPAD4 or rmPAD2 and citrullinated residues were determined by proteolytic digestion followed by mass spectrometry. The positions of the citrullination sites are schematically depicted in the three polypeptide chains of fibrinogen: hPAD2, filled circles; hPAD4, open circles; rmPAD2, filled triangles. The grey boxes at the N-termini of the fibrinogen chains represent signal peptides (for all chains) and fibrinopeptides (for the α- and β-chains). Beneath each fibrinogen chain (α, β or γ), previously detected citrullination sites have been depicted; hPAD2, filled circles; hPAD4, open circles [15,16]. Amino acid numbering is relative to the start of the signal peptides.

To obtain a comprehensive overview of the residues in human fibrinogen that are citrullinated by hPAD2 and hPAD4, citrullinated fibrinogen was proteolytically digested with three different enzymes (trypsin, chymotrypsin, GluC), to optimize the sequence coverage and to eliminate the influence of citrullination on cleavage. All digests were analyzed by LC-MS/MS using both CID and ETD fragmentation to obtain as much sequence information as possible. This allowed the identification of 42 citrullinated residues, 27 of which were found in the α-chain, 11 in the β-chain and 4 in the γ-chain of human fibrinogen (Figure 3). The total coverage of the mature fibrinogen polypeptide sequences obtained by this approach was similar in all experiments, approximately 91% for the α-chain, 86% for the β-chain and 89% for the γ-chain. The majority of the citrullination sites were identified in fibrinogen treated with hPAD2. In the α-chain, 27 arginines were deiminated by hPAD2, 17 of which were also citrullinated by hPAD4. No arginines that were specifically deiminated by hPAD4 were detected. In the β-chain, six arginine residues were deiminated by both hPAD2 and hPAD4, three only by hPAD2 and two only by hPAD4. Finally, in the γ-chain four citrullination sites were identified, three for each of the two PADs, two of which were citrullinated by both enzymes. A similar analysis of residues of human fibrinogen that are citrullinated by rmPAD2 was performed, which allowed the identification of 40 citrullinated residues, 23 in the α-chain, 13 in the β-chain and 4 in the γ-chain. Although the majority of these residues was also deiminated by hPAD2 (17 in the α-chain, 7 in the β-chain and 2 in the γ-chain), some deiminated residues were specifically detected in either the rmPAD2- or hPAD2-treated material (Figure 2), which might be related either to PAD specificity or undersampling by the mass spectrometer.

### Generation of citrullinated human fibrinogen peptides

To investigate which of the identified citrullinated residues in human fibrinogen are important for the recognition by autoantibodies in RA sera, *in vitro *citrullinated fibrinogen was proteolytically digested and fractionated by ion exchange chromatography. To this end, fibrinogen was citrullinated with rmPAD2, because the specific activity of this enzyme is higher than that of hPAD2 and, as a consequence, the amount of PAD protein needed was lower. Citrullinated fibrinogen was in this case digested with either trypsin (T), chymotrypsin (C) or Lys-N (L) to generate different pools of citrullinated peptides [24].

The individual digests were separated using SCX chromatography followed by mass spectrometry to identify the citrullinated peptides present in the different fractions. Adjacent fractions with largely overlapping peptide compositions were pooled (Supplementary Figure S1 in Additional file 1), giving a total of 29 fractions with different sets of citrullinated fibrinogen peptides that were selected for *i*SPR analysis. The citrullinated peptide composition of these fractions is shown in Supplementary Tables S1 (trypsin), S2 (Lys-N) and S3 (chymotrypsin) in Additional file 1.

### Recognition of citrullinated fibrinogen peptide fractions by RA sera

**Figure 4:**
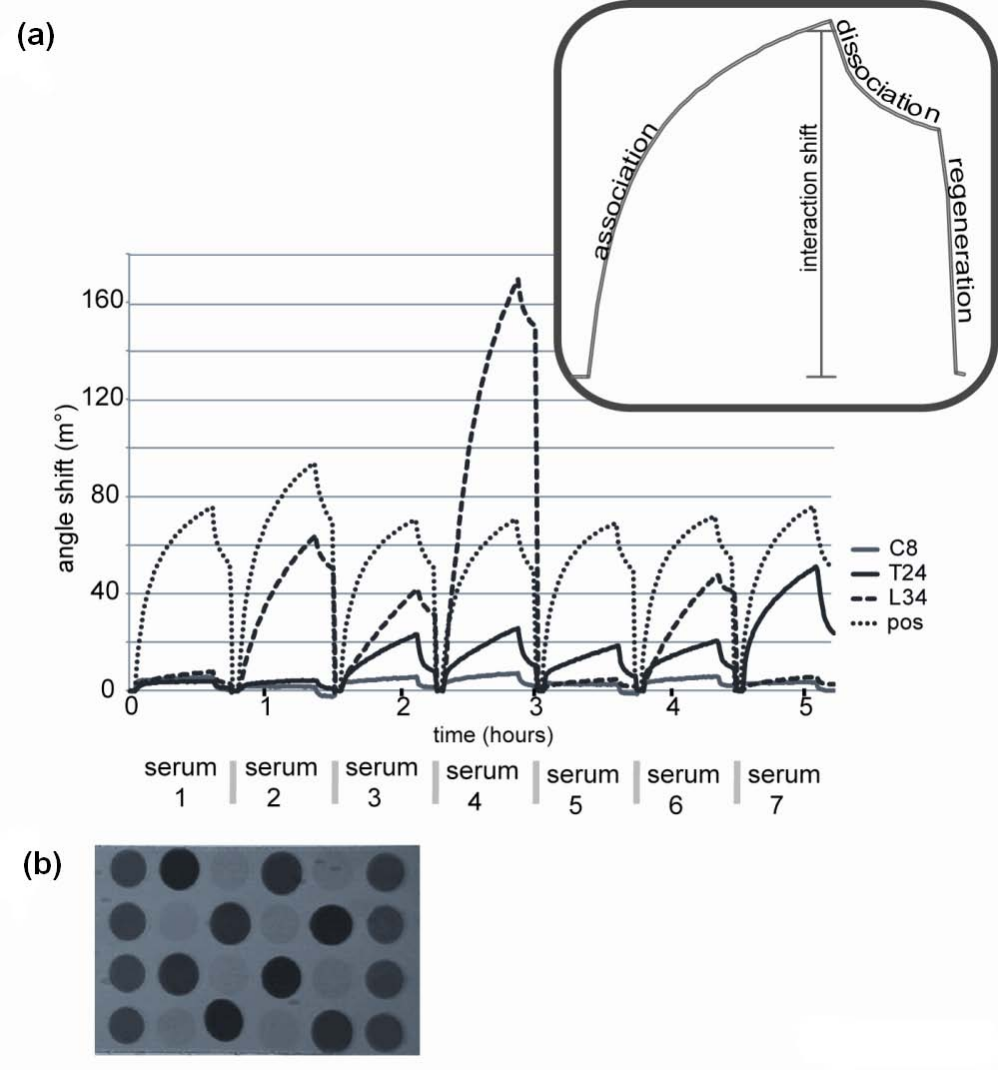
**Analysis of autoantibody reactivity in patient sera by *i*SPR**. **(a) **Sensorgrams showing the reactivity of seven sera to citrullinated peptides. The reactivity of seven RA sera with four different spots on the array was determined by *i*SPR monitoring. These spots contained either citrullinated fibrinogen fractions (C8, L34 or T24) or a positive control peptide (pos). In the sensorgrams the angle shifts in millidegrees (m°) are plotted as a function of time. **(b) **Reconstructed image of an *i*SPR sensorchip showing the resonance at 24 spots at a certain angle and a certain timepoint during scanning of the array.

The (pooled) SCX fractions were immobilized in discrete spots on the surface of *i*SPR sensor discs and the reactivity of 98 anti-CCP-positive and 4 anti-CCP-negative RA sera with these fractions was monitored by *i*SPR measurements, which we previously have shown to represent a suitable system to detect autoantibody reactivities against multiple targets simultaneously [22,23]. Data generated with *i*SPR can be depicted in a sensorgram, in which the resonance angle shift is plotted against time. An example of a sensorgram generated with this material is depicted in Figure 4, which illustrates the reactivity of seven RA sera with three fibrinogen peptide fractions (C8, L34 and T24) and with a synthetic citrullinated peptide, which was used as a positive control. The (resonance) angle shift resulting from the association of serum antibodies to peptides on the sensor disc surface was determined for each serum and each spot (Figure 4a, inset). The use of 24 spots per chip (Figure 4b) allowed the simultaneous analysis of multiple fractions, strongly reducing the total time required for the experiment and increased the throughput of patient sera. As a total of 29 fractions were analyzed, two chips, together containing all fractions as well as positive controls, were analyzed with all sera. The results clearly show that the efficiency by which specific fractions were recognized strongly differs between individual sera. For example, serum 1 in Figure 4a is not reactive with any of the citrullinated fibrinogen peptides, although it clearly contains antibodies recognizing the positive control peptide, whereas serum 4 is clearly reactive with at least two of the citrullinated fibrinogen fractions.

We decided to classify the fibrinogen peptide fractions based upon their reactivity with patient sera as defined by angle shifts above a well-defined cutoff value and upon the percentage of sera that showed reactivity (see Methods). Nine of the 29 fractions were strongly recognized by RA sera, while an additional 9 fractions showed moderate levels of reactivity with RA sera (Table 1).

**Table 1:** Recognition of citrullinated fibrinogen peptide-containing fractions by RA sera

Strongly reactive fractions^a^	% reactive sera	Moderately reactive fractions^b^	% reactive sera	Non reactive fractions^c^
T32	75	T31	54	C12
T27	57	L34	47	C14
C8	51	C10	44	C22
T28	51	L33	43	C25
T26	50	C27	36	C31
L37	46	L27	36	C34
L28	39	C29	33	L24
T29	39	C28	30	L26
T24	36	C30	25	L29
				L30
				L31

^a ^Fractions that are recognized by at least 25% of the RA sera with reactivities exceeding a cut-off value of the mean plus 8 times the standard deviation of anti-CCP-negative sera. T, C and L refer to the proteases used to fragment citrullinated fibrinogen; numbers correspond to fraction numbers of the fractionation by cation exchange chromatography.

^b ^Fractions that are recognized by at least 25% of the RA sera with reactivities exceeding a cut-off value of the mean plus two times the standard deviation of anti-CCP-negative sera.

^c^The remaining fractions that were analysed.

RA, rheumatoid arthritis.

**Figure 5:**
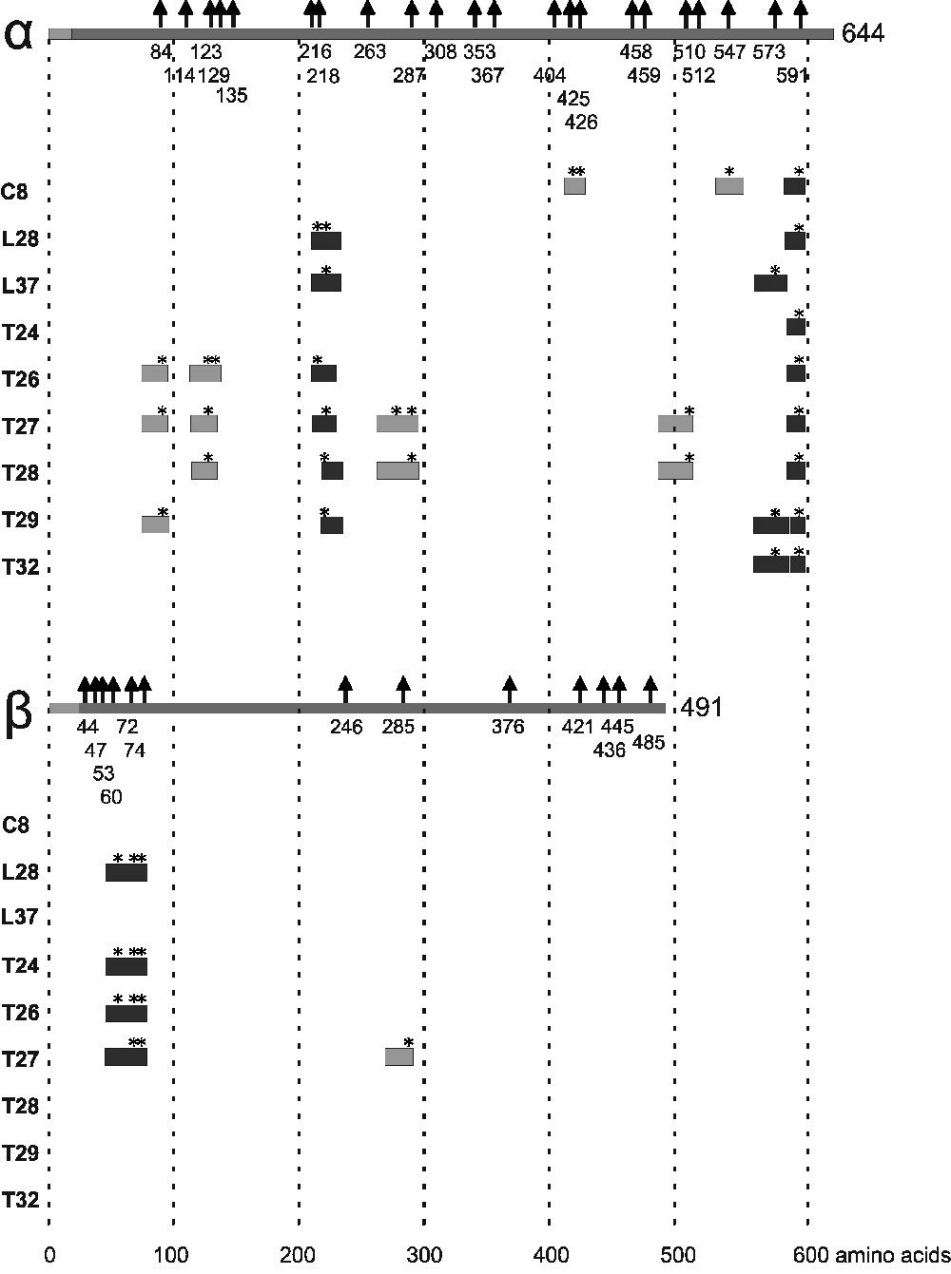
**Mapping of major citrullinated fibrinogen epitopes recognized by RA sera**. The citrullinated peptides present in the most strongly reactive fractions are schematically aligned with the α- and β-chains of fibrinogen. Asterisks indicate the citrullinated residues in the peptides. Citrullinated peptides (obtained by *in vitro *citrullination with rmPAD2) that are present in the most reactive fractions of at least two of the three proteolytic digests are highlighted in dark grey, whereas the other peptides in these fractions are indicated with the light grey boxes.

The citrullinated peptides present in the strongly reactive fractions are schematically aligned with the fibrinogen α- and β-chains in Figure 5. This shows that the most efficiently recognized citrullinated peptides correspond to three regions of human fibrinogen. The citrullinated residues present in these regions are 216, 218, 573 and 591 in the α-chain and 60, 72 and 74 in the β-chain (Supplementary Table S4 in Additional file 1).

## Discussion

In this paper we have applied a novel method to map citrullinated epitopes of human fibrinogen that are recognized by RA patient sera. This method is widely applicable, not only for epitopes generated by post-translational modifications, but also for epitopes consisting of only standard amino acids. Three regions, two in the α- and one in the β-chain, containing major epitopes for RA-autoantibodies were identified. The presence of citrullinated fibrinogen in the inflamed synovium of rheumatoid arthritis patients [1] and the detection of RA-associated autoantibodies against citrullinated fibrinogen imply that this protein is a genuine autoantigen in RA [4] and previous studies have already demonstrated that the citrullination of fibrinogen is complex, with deiminated arginines on all three chains and at various positions within these chains. Our combined data identified 54 arginine residues that can be deiminated by hPAD2, hPAD4 or rmPAD2 (Figure 3 and Supplementary Figure S2 in Additional file 1). Thirty-two of these are associated with the α-chain, 17 with the β-chain and 5 with the γ-chain. All previously reported citrullination sites were found in our study (Figure 3) [15,16]. In addition, 26 novel citrullination sites were identified, 12 in the α-chain (positions 69, 84, 114, 135, 181, 186, 216, 218, 263, 353, 443 and 458), 10 in the β-chain (positions 47, 224, 246, 285, 376, 410, 421, 436, 445 and 485) and 4 in the γ-chain (positions 40, 223, 282 and 417).

In total, 54 of the 81 arginines (66%) in human fibrinogen were found to be susceptible to citrullination (Supplementary Figure S2 in Additional file 1). When comparing individual PADs, both hPAD2 and rmPAD2 deiminate 39 residues, whereas 28 residues can be citrullinated by hPAD4. The higher number of residues citrullinated by PAD2 compared to PAD4 is in agreement with observations that the amino acid context of the peptidylarginine is more important for PAD4 than for PAD2 (manuscript in preparation; [25]).

The digestion of citrullinated fibrinogen with three distinct proteases to analyze autoantibody reactivities with the fractionated peptides implied that a large part of the (citrullinated) protein was covered by peptides suitable for both LC-MS/MS analysis and immobilization. An important advantage of *i*SPR is that it can be used to monitor the reactivity of patient sera with multiple antigenic molecules simultaneously, which is less time-consuming and more accurate than other current methods to screen sera, such as ELISA. The *i*SPR results obtained with the fractionated proteolytic digests of fibrinogen revealed that RA sera are reactive with specific regions of citrullinated fibrinogen. These regions are present in the fibrinogen sequence around the citrullinated residues at position 216, 218, 573 and 591 of the α-chain and at positions 60, 72 and 74 of the β-chain. Previously, several studies have reported the reactivity of RA sera with citrullinated epitopes of fibrinogen. The data related to the epitopes identified in our study are summarized in Table 2[4,7,9,15,16]. Although some overlap between these data is observed, also clear differences are present, which may at least in part be due to the differences in the peptides that were used and the procedures that were applied to immobilize these peptides. Using synthetic peptides the N-terminal region of the α-chain of human fibrinogen (with citrullinated residues at positions 38 and 42) was observed to be frequently targeted by RA sera [4]. Because these residues were not detectably citrullinated by rmPAD2, our data do not allow conclusions concerning the recognition of this region of fibrinogen by RA sera. In contrast, citrullination of the arginine residues at positions 216 and 218 of the α-chain have not been reported in previous studies, in which fibrinogen was citrullinated by hPAD2 or hPAD4 (Figure 3) [15,16], which is most likely due to the inclusion of rmPAD2 in the present study. The poor recognition of synthetic peptides comprising this region of fibrinogen by RA sera in previous studies [4,9] might be explained by differences in the presentation of the epitope(s) by (partially) different peptides. Currently we are using synthetic citrullinated peptides derived from the data presented in Figure 5 to verify the presence of major epitopes targeted by RA autoantibodies at these positions in fibrinogen and to investigate their diagnostic and prognostic value in more detail.

**Table 2:** Citrullinated fibrinogen epitopes detected also in previous studies

Reference	**Peptide used**^**a**^	Citrullination sites	Fibrinogen chain	Percentage of reactive sera (%)
Sebbag *et al*. [4].	**___________R**D**R**QHLPLIKMKPVP	216, 218	α	0
	_________LPS**R**D**R**QHLPLIKMK	216, 218	α	0
Perez *et al*. [9].	____IAKDLLPS**R**DRQHLPLIK	216	α	18
	____IAKDLLPSRD**R**QHLPLIK	218	α	0
	____IAKDLLPS**R**D**R**QHLPLIK	216, 218	α	0
Sebbag *et al*. [4].	_____________SSHHPGIAEFP**R**GK	573	α	25
Kubota *et al*. [15].	___SGIFTNTKESSSHHPGIAEFP**R**GKSSSY	573	α	n.d.
Nakayama-Hamada *et al*. [16].	b)	573	α	n.d.
Sebbag *et al*. [4].	**_____________R**GDSTFESKSYKMAD	591	α	0
	__________SYN**R**GDSTFESKSYK	591	α	20
Kubota *et al*. [15].	____QFTSSTSYN**R**GDSTFESKS	591	α	n.d.
Nakayama-Hamada *et al*. [16].	b)	591	α	n.d.
Perez *et al*. [9].	A**R**GH**R**PLDKK**R**EEAPSL**R**PA	44, 47, 53, 60	β	3
	ARGH**R**PLDKK**R**EEAPSL**R**PA	47, 53, 60	β	9
	A**R**GHRPLDKK**R**EEAPSL**R**PA	44, 53, 60	β	6
Sebbag *et al*. [4].	_______DKK**R**EEAPSL**R**PAPP	53,60	β	0
Perez *et al*. [9].	ARGHRPLDKK**R**EEAPSL**R**PA	53,60	β	12
	ARGHRPLDKKREEAPSL**R**PA	60	β	12
	A**R**GHRPLDKKREEAPSL**R**PA	44, 60	β	0
	ARGH**R**PLDKKREEAPSL**R**PA	47, 60	β	36
	A**R**GH**R**PLDKKREEAPSL**R**PA	44, 47, 60	β	12
	**______________R**ARPAKAAATQKKVERKA	72	β	0
	______________RA**R**PAKAAATQKKVERKA	74	β	0
Sebbag *et al*. [4].	**__R**PAPPPISGGGY**R**A**R**	60, 72, 74	β	28
Hill *et al*. [7].	___________GGY**R**A**R**PAKAAAT	72, 74	β	0
Perez *et al*. [9].	**_____________R**A**R**PAKAAATQKKVERKA	72, 74	β	0
Nakayama-Hamada *et al*. [16].	b)	53, 60, 72, 74	β	n.d.
Perez *et al*. [9].	**_____________R**ARPAKAAATQKKVE**R**KA	72, 87	β	0
	_____________RA**R**PAKAAATQKKVE**R**KA	74, 87	β	0
	**_____________R**A**R**PAKAAATQKKVE**R**KA	72, 74, 87	β	0

^a) ^Arginines that can be citrullinated by either hPAD2, hPAD4 or rmPAD2 are highlighted in bold.

^b) ^Nakayama-Hamada and colleagues only showed the identified citrullination sites and not which peptides containing the sites were found.

n.d.: not determined

The citrullinated epitopes of fibrinogen reactive with RA sera are particularly relevant for diagnostic applications, since citrullinated human fibrinogen displays a sensitivity and specificity very similar to the widely used anti-CCP test for ACPA detection [10]. The use of individual citrullinated peptides in a multiplex assay, however, enables a better quality control of diagnostic test performance and the results of this study indeed can be used to rationally design the peptides for such multiplex systems. The need for multiplex ACPA analysis systems has recently increased by the observations that the so-called fine-specificity of these antibodies has a predictive value for the way the disease will progress [26,27]. It would be particularly interesting to analyze the ACPA response to the citrullinated fibrinogen epitopes in various stages of (early) RA and to compare the reactivity profiles with clinical features, which may provide opportunities for more personalized treatment.

Knowledge on the citrullinated autoepitopes targeted by the antibodies in the inflamed synovia of RA patients is also important for a better understanding of the pathophysiology, because the resulting immune complexes may directly contribute to the progression and chronicity of the disease [28].

## Conclusions

A new method was developed for the comprehensive analysis of epitopes that are formed by post-translational modifications using the (enzymatically modified) protein of interest as starting material. This method, in which chromatographic fractionation of proteolytic digests is combined with mass spectrometry and surface plasmon resonance imaging on microarrays, allows the simultaneous mapping of modified residues and epitopes. Using this method, fifty four citrullination sites and three citrullinated epitope regions frequently recognized by ACPA in the sera of RA patients were identified in human fibrinogen, one of the main autoantigenic proteins in this disease.

## Abbreviations

ACPA: anti-citrullinated protein antibodies; CCP: cyclic citrullinated peptide; CID: collison induced dissociation; EDC: N-ethyl-N'-(dimethylaminopropyl) carbodiimide hydrochloride; ETD: electron transfer induced dissociation; FTICR: Fourier transform ion cyclotron resonance; HPLC: high-performance liquid chromatography; *i*SPR: surface plasmon resonance imaging; LC-MS/MS: liquid chromatography - tandem mass spectrometry; LTQ: linear trap quadrupole; NHS: N-hydroxysuccinimide; PAD: peptidylarginine deiminase; RA: rheumatoid arthritis; RF: rheumatoid factor; rmPAD2: rabbit muscle PAD; SCX: strong cation exchange.

## Competing interests

The authors declare that they have no competing interests

## Authors' contributions

JvB carried out protein modifications, performed the data analysis, and helped to draft the manuscript. RR participated in the design of the study, carried out the chromatography and mass spectrometry analyses, and contributed to writing of the manuscript. LEA carried out iSPR analyses and was involved in data analysis. JSV participated in protein modification and characterization. AL participated in the design of the study and the iSPR analyses. AH contributed to the design of the study and the analysis of mass spectrometry data. RS and GP conceived of the study, participated in the design of the study and in the writing of the manuscript. All authors contributed to the interpretation of the data and all authors read and approved the final manuscript.

## Supplementary Material

Additional file 1 **Supplementary Tables S1 to S4 and Supplementary Figures S1 to S2**. Supplementary Table S1: Peptide composition of SCX fractions of citrullinated fibrinogen digested with trypsin. Supplementary Table S2: Peptide composition of SCX fractions of citrullinated fibrinogen digested with Lys-N. Supplementary Table S3: Peptide composition of SCX fractions of citrullinated fibrinogen digested with chymotrypsin. Supplementary Table S4: Citrullinated fibrinogen peptides containing the major epitopes present in the reactive fractions. Supplementary Figure S1: Chromatographic fractionation of proteolytically digested citrullinated fibrinogen. Supplementary Figure S2: Amino acid sequence of the three human fibrinogen chains. Click here for file
