# Supplementary material for: Mapping of citrullinated fibrinogen B-cell epitopes in rheumatoid arthritis by imaging surface plasmon resonance
Source: Arthritis Res Ther. 2010 Dec 23;12(6):R219. doi: 10.1186/ar3205 (PMC3046532; doi:10.1186/ar3205)
Supplement: Additional file 1 — Supplementary Tables S1 to S4 and Supplementary Figures S1 to S2. Supplementary Table S1: Peptide composition of SCX fractions of citrullinated fibrinogen digested with trypsin. Supplementary Table S2: Peptide composition of SCX fractions of citrullinated fibrinogen digested with Lys-N. Supplementary Table S3: Peptide composition of SCX fractions of citrullinated fibrinogen digested with chymotrypsin. Supplementary Table S4: Citrullinated fibrinogen peptides containing the major epitopes present in the reactive fractions. Supplementary Figure S1: Chromatographic fractionation of proteolytically digested citrullinated fibrinogen. Supplementary Figure S2: Amino acid sequence of the three human fibrinogen chains. [file ar3205-S1.DOC]

**Supplementary material**

**Supplementary Table 1.** Peptide composition of SCX fractions of citrullinated fibrinogen digested with trypsin.

Light and dark gray shading indicates pooled fractions, with in bold the indication of the pools that were used for spotting on the microarrays. Citrullinated (R) and deamidated (N) amino acids are underlined.

**Supplementary Table 2.** Peptide composition of SCX fractions of citrullinated fibrinogen digested with Lys-N.

Light and dark gray shading indicates pooled fractions, with in bold the indication of the pools that were used for spotting on the microarrays. Citrullinated (R) and deamidated (N) amino acids are underlined.

**Supplementary Table 3.** Peptide composition of SCX fractions of citrullinated fibrinogen digested with chymotrypsin.

Light and dark gray shading indicates pooled fractions, with in bold the indication of the pools that were used for spotting on the microarrays. Citrullinated (R) and deamidated (N) amino acids are underlined.

**Supplementary Table 4.** Citrullinated fibrinogen peptides containing the major epitopes present in the reactive fractions.

| **Fibrinogen chain** | **Fraction number** | **Percentage of reactive RA sera (%)**^a^ | **Peptide**^b^ | **Citrullination site(s)** |
| --- | --- | --- | --- | --- |
| α | T26 | 50 | DLLPSRDR | 216 |
|  | T27 | 57 | DLLPSRDR | 218 |
|  | L37 | 46 | KDLLPSRDRQHLPLI |  |
|  | T28 | 51 | DRQHLPLIK |  |
|  | T29 | 39 |  |  |
|  | L28 | 39 | KDLLPSRDRQHLPLI | 216,218 |
|  | T28 | 51 | ESSSHHPGIAEFPSRGK | 573 |
|  | T29 | 39 |  |  |
|  | T32 | 75 |  |  |
|  | L37 | 46 | KESSSHHPGIAEFPSRG |  |
|  | C8 | 51 | TSSTSYNRGDSTF | 591 |
|  | L28 | 39 | KQFTSSTSYNRGDSTFES |  |
|  | L37 | 46 |  |  |
|  | L28 | 39 | KQFTSSTSYNRGDSTFESKSY |  |
|  | T24 | 36 | QFTSSTSYNRGDSTFESK |  |
|  | T26  T32 | 50  75 |  |  |
| β | L28 | 39 | KREEAPSLRPAPPPISGGGYRARPA | 53,72,74 |
|  | T24 | 36 | EEAPSLRPAPPPISGGGYRARPAK | 60, 72, 74 |
|  | T26 | 50 |  |  |
|  | T27 | 57 | EEAPSLRPAPPPISGGGYRARPAK | 72,74 |
|  |  |  |  |  |

^a^ The percentage of sera showing reactivities exceeding a cut-off value of the mean plus 2 times the standard deviation.

^b^ An overview of the complete list of peptides found in each fraction is provided in Supplementary Tables 1 - 3.

**Supplementary Figure 1.** Chromatographic fractionation of proteolytically digested citrullinated fibrinogen. Citrullinated fibrinogen digested with trypsin, Lys-N or chymotrypsin was fractionated by strong cation exchange (SCX) chromatography. The fibrinogen peptide containing fractions were pooled as indicated.

**
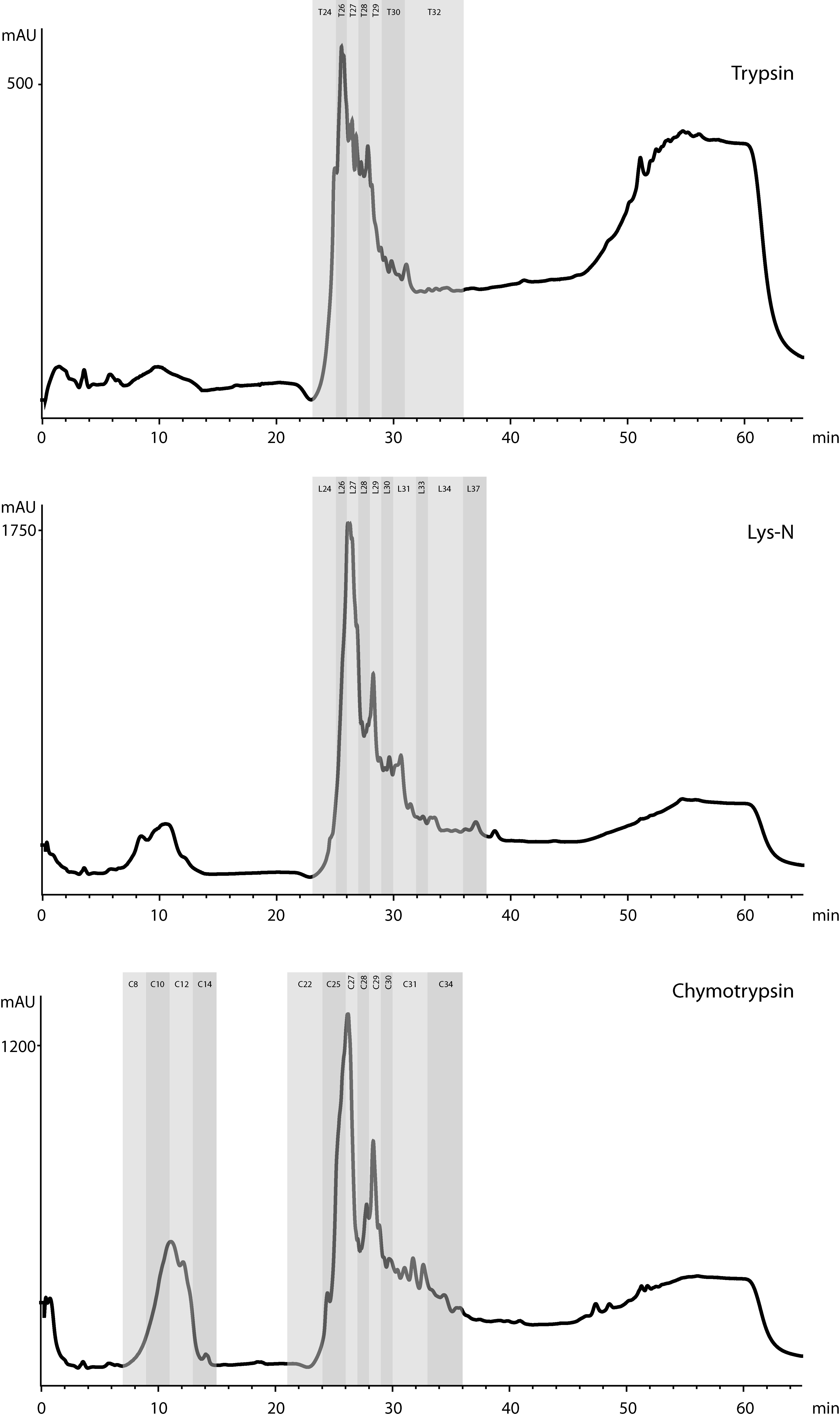

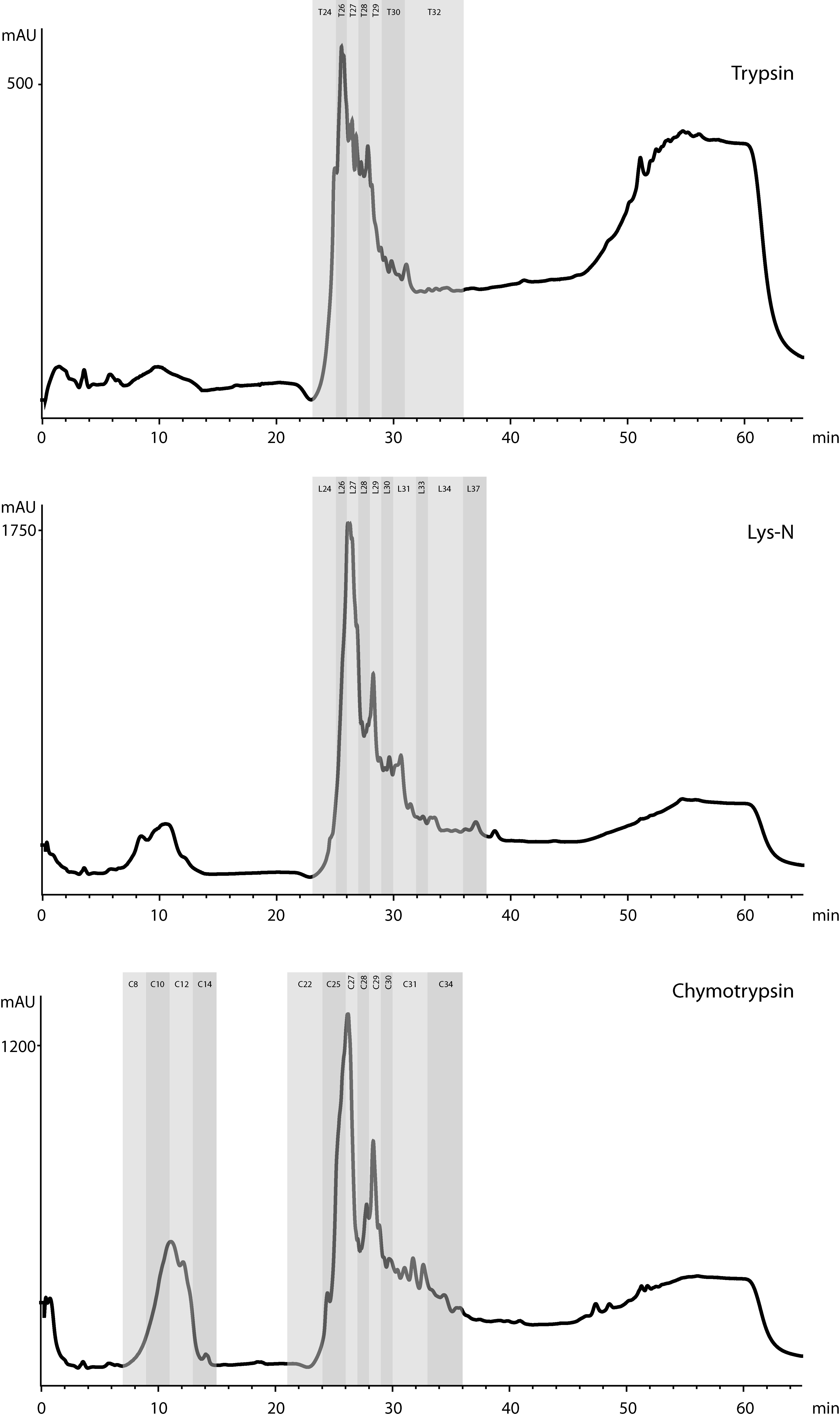

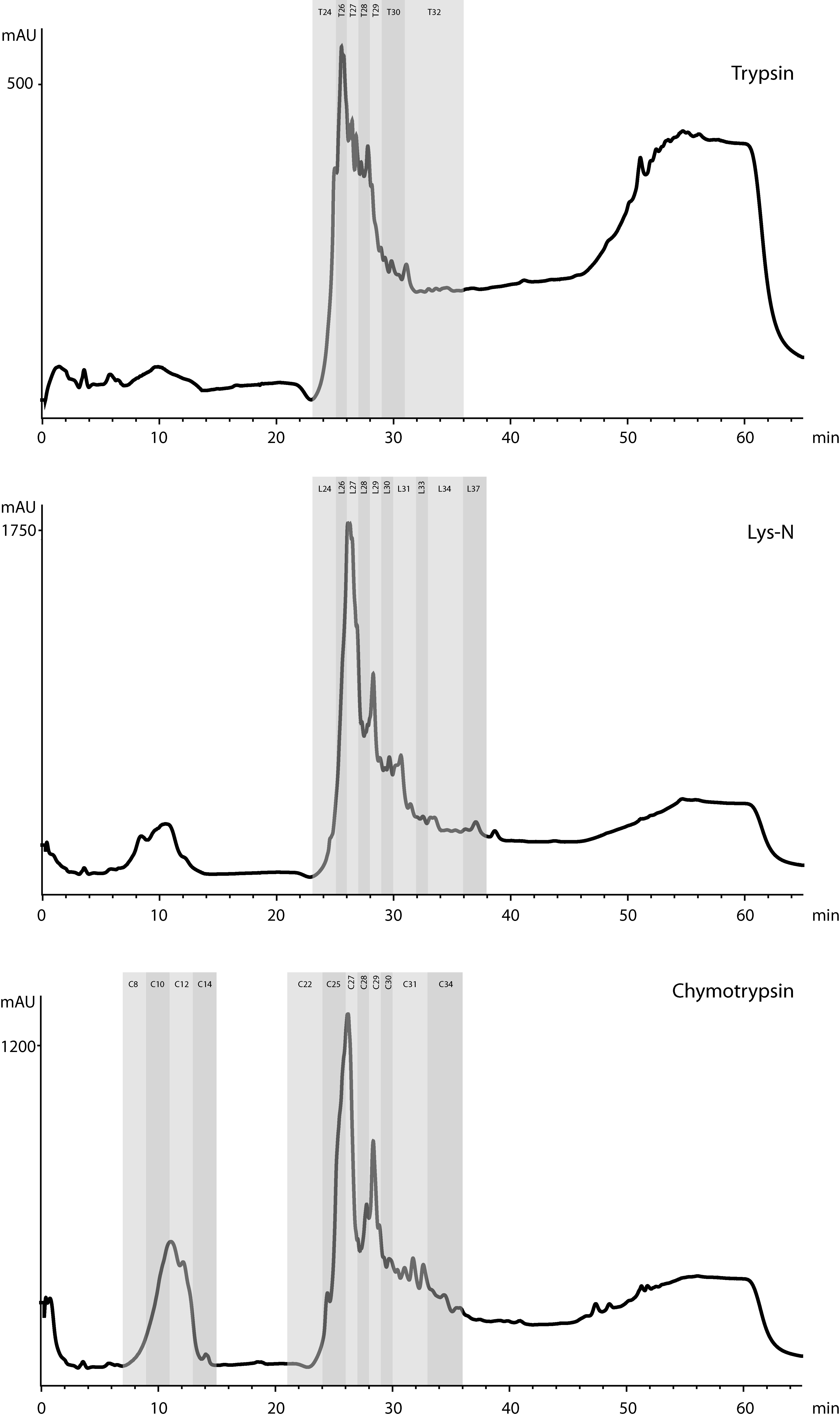
**

|  |
| --- |

**Supplementary Figure 2.** Amino acid sequence of the three human fibrinogen chains. Arginine residues are highlighted; arginines that can be citrullinated by either hPAD2, hPAD4 or rmPAD2 are highlighted with the black boxes.

*Fibrinogen α-chain*

**1** MFSMRIVCLV LSVVGTAWTA DSGEGDFLAE GGGVRGPRVV ERHQSACKDS DWPFCSDEDW

**61** NYKCPSGCRM KGLIDEVNQD FTNRINKLKN *S*LFEYQKNNK DSHSLTTNIM EILRGDFSSA

**121** NNRDNTYNRV SEDLRSRIEV LKRKVIEKVQ HIQLLQKNVR AQLVDMKRLE VDIDIKIRSC

**181** RGSWSRALAR EVDLKDYEDQ QKQLEQVIAK DLLPSRDRQH LPLIKMKPVP DLVPGNFKSQ

**241** LQKVPPEWKA LTDMPQMRME LERPGGNEIT RGGSTSYGTG SETESPRNPS SAGSWNSGSS

**301** GPGSTGNRNP GSSGTGGTAT WKPGSSGPGS AGSWNSGSSG TGSTGNQNPG SPRPGSTGTW

**361** NPGSSERGSA GHWTSESSVS GSTGQWHSES GSFRPDSPGS GNARPNNPDW GTFEEVSGNV

**421** SPGTRREYHT EKLVTSKGDK ELRTGKEKVT SGSTTTTRRS CSKTVTKTVI GPDGHKEVTK

**481** EVVTSEDGSD CPEAMDLGTL SGIGTLDGFR HRHPDEAAFF DTASTGKTFP GFFSPMLGEF

**541** VSETESRGSE SGIFTNTKES SSHHPGIAEF PSRGKSSSYS KQFTSSTSYN RGDSTFESKS

**601** YKMADEAGSE ADHEGTHSTK RGHAKSRPVR GIHTSPLGKP SLSP

*Fibrinogen β-chain*

**1** MKRMVSWSFH KLKTMKHLLL LLLCVFLVKS QGVNDNEEGF FSARGHRPLD KKREEAPSLR

**61** PAPPPISGGG YRARPAKAAA TQKKVERKAP DAGGCLHADP DLGVLCPTGC QLQEALLQQE

**121** RPIRNSVDEL NNNVEAVSQT SSSSFQYMYL LKDLWQKRQK QVKDNENVVN EYSSELEKHQ

**181** LYIDETVNSN IPTNLRVLRS ILENLRSKIQ KLESDVSAQM EYCRTPCTVS CNIPVVSGKE

**241** CEEIIRKGGE TSEMYLIQPD SSVKPYRVYC DMNTENGGWT VIQNRQDGSV DFGRKWDPYK

**301** QGFGNVATNT DGKNYCGLPG EYWLGNDKIS QLTRMGPTEL LIEMEDWKGD KVKAHYGGFT

**361** VQNEANKYQI SVNKYRGTAG NALMDGASQL MGENRTMTIH NGMFFSTYDR DNDGWLTSDP

**421** RKQCSKEDGG GWWYNRCHAA NPNGRYYWGG QYTWDMAKHG TDDGVVWMNW KGSWYSMRKM

**481** SMKIRPFFPQ Q

*Fibrinogen γ-chain*

**1** MSWSLHPRNL ILYFYALLFL SSTCVAYVAT RDNCCILDER FGSYCPTTCG IADFLSTYQT

**61** KVDKDLQSLE DILHQVENKT SEVKQLIKAI QLTYNPDESS KPNMIDAATL KSRIMLEEIM

**121** KYEASILTHD SSIRYLQEIY NSNNQKIVNL KEKVAQLEAQ CQEPCKDTVQ IHDITGKDCQ

**181** DIANKGAKQS GLYFIKPLKA NQQFLVYCEI DGSGNGWTVF QKRLDGSVDF KKNWIQYKEG

**241** FGHLSPTGTT EFWLGNEKIH LISTQSAIPY ALRVELEDWN GRTSTADYAM FKVGPEADKY

**301** RLTYAYFAGG DAGDAFDGFD FGDDPSDKFF TSHNGMQFST WDNDNDKFEG NCAEQDGSGW

**361** WMNKCHAGHL NGVYYQGGTY SKASTPNGYD NGIIWATWKT RWYSMKKTTM KIIPFNRLTI

**421** GEGQQHHLGG AKQAGDV
